# The associations between physical activity, microbiome and metabolic adaptation in sedentary overweight adults

**DOI:** 10.3389/fnut.2025.1722274

**Published:** 2026-02-02

**Authors:** Eylam Ziv Av, Alisa Greenberg, Tzachi Knaan, Edward L. Melanson, Ilan Youngster, Gal Dubnov-Raz, Elhanan Borenstein, Yftach Gepner

**Affiliations:** 1 Department of Health Promotion, Faculty of Medical & Health Sciences, and Sylvan Adams Sports Institute, School of Public Health, Tel Aviv University, Tel Aviv, Israel; 2 Department of Clinical Microbiology and Immunology, Gray Faculty of Medical & Health Sciences, Tel Aviv University, Tel Aviv, Israel; 3 Division of Endocrinology, Metabolism and Diabetes, Department of Medicine, University of Colorado Anschutz Medical Campus, Aurora, CO, United States; 4 Division of Geriatric Medicine, Department of Medicine, The University of Colorado Anschutz Medical Campus, Aurora, CO, United States; 5 Pediatric Division and Center for Microbiome Research, Shamir Medical Center, Be’er Ya’akov, Israel; 6 Gray Faculty of Medical & Health Sciences, Tel Aviv University, Tel Aviv, Israel; 7 Faculty of Medicine, National Institute for Sports Medicine, Sheba Medical Center, Tel HaShomer, Tel Aviv, Israel; 8 Pediatric Exercise and Lifestyle Clinic, Safra Children’s Hospital, Sheba Medical Center, Tel HaShomer, Ramat Gan, Israel; 9 The Blavatnik School of Computer Science and AI, Tel Aviv University, Tel Aviv, Israel; 10 Santa Fe Institute, Santa Fe, NM, United States

**Keywords:** body composition, gut microbiome, metabolic adaptation, obesity, physical activity

## Abstract

Despite well-established benefits of exercise on metabolic regulation and the gut microbiome (GM), its impact on body composition is inconsistent and often attenuated by metabolic adaptation. This compensation mechanism adjusts energy expenditure including total daily energy expenditure (TDEE) and resting metabolic rate (RMR). Intra-individual variation in exercise response remains unclear, but might be explained by the GM. In this well-controlled study, we investigated the relationship between aerobic exercise, GM composition, and metabolic adaptation in a cohort of 16 sedentary overweight adults (ages 21–45, 50% female) over a 12-week moderate-intensity intervention (65–75% HRmax; 20 kcal/kg/week). Pre- and post-intervention RMR was measured via whole-room calorimetry, TDEE by doubly labeled water, and GM composition via shotgun metagenomics. While body composition did not change at the group-level, a subset of participants (“responders”) showed improved body composition and aerobic capacity. Using machine learning, we identified bacterial species, including *Faecalibacterium prausnitzii* species, whose abundance pre-training is predictive of response. Additionally, we found that responder GM communities are more compositionally cohesive and post-training increases in GM diversity are associated with higher TDEE and RMR. These findings highlight the complex interaction between exercise, metabolism and the GM, and suggest that baseline GM characteristics may contribute to individual variability in metabolic adaptation. This insight may help guide microbiome-informed strategies to enhance exercise efficacy.

Clinical trial registration: ClinicalTrials.gov, identifier NCT04460040.

## 1 Introduction

Global obesity rates have increased sharply over the past decades, reaching 10.8% in men and 14.9% in women (1), and is linked to various comorbidities, including diabetes, hypertension, and fatty liver disease (2). While obesity is defined by body mass index (BMI), recent research, suggests that body composition, quantified by fat mass (FM) and fat-free mass (FFM), may better reflect the physiological state and health risks (3). A key driver of obesity is a positive energy balance, influenced by behaviors like exercise and sedentary time, and physiological factors like resting metabolic rate (RMR) (4, 5). While the traditional additive model suggests a linear relationship between exercise and total daily energy expenditure (TDEE) (6), recent studies propose a constrained model, where the body adapts to increased exercise by reducing energy spent on other activities (7). This metabolic adaptation results in smaller-than-expected increase in TDEE following additional exercise (8), leading to weight loss outcomes that are lower than predicted by the additive model (9–13). The constrained model may clarify long-term studies indicating minimal weight loss despite high exercise intensity, fueling the ongoing debate on the effectiveness of structured exercise as a weight loss strategy.

Recent research highlights the importance of the human microbiome, particularly the gut microbiome (GM), in shaping human health (14) through regulation of host digestion, immune defense, toxin processing, and compound synthesis (15). Disruption of the GM balance has been linked to several chronic conditions such as inflammatory bowel diseases, diabetes, and obesity (16). *Faecalibacterium prausnitzii* (*F. prausnitzii*), a key gut microbe, plays a crucial role in human health through its anti-inflammatory effects, partly due to metabolites like butyrate, which inhibit NF-κB activation and IL-8 production (17, 18).

Given the GMs critical role in human health, there is a growing interest in how lifestyle factors influence its dynamics. While the impact of diet is well-established, our understanding of the interplay between the GM and exercise remains limited. Emerging evidence suggests that exercise to enhance positive GM phenotypes, including increased abundance of species associated with intestinal health and elevated microbial diversity (19). Similarly, mouse models have revealed that aerobic exercise elevates bacterial populations associated with leanness (20), and in humans, athletes and active individuals exhibit higher taxonomic diversity than healthy sedentary individuals, with increased presence of beneficial species such as *F. prausnitzii* and *Akkermansia muciniphila* (21–23). A longitudinal study has suggested clear but reversible effects of exercise on GM, highlighting its transient influence (24). These findings support a role for exercise as a modifiable lifestyle factor that may help steer the GM toward anti-inflammatory and metabolically favorable configurations, potentially enhancing host metabolic function via microbial metabolite signaling and energy homeostasis.

The GM is an important environmental factor of energy balance, regulating both energy intake and expenditure, though its relationship with the host energy expenditure remains uncertain (25). Research has indicated, for example, a GM involvement in host metabolism, the modulation of energy storage and lipid metabolism (26, 27). Studies in rodents and humans have suggested correlations between the GM composition, RMR, and FM percentage (28, 29). However, quantifying the direct contribution of the GM to host energy expenditure is challenging due to the anaerobic nature of this environment. Consequently, studies using indirect calorimetry, in-room calorimetry, and TDEE measurements are needed, but remain scarce due to experimental and measurement challenges (30). In this study, we aim to investigate whether moderate aerobic exercise induces specific changes in gut microbiome composition that are associated with interindividual variability in metabolic adaptation.

We hypothesize that individuals who exhibit attenuated increases in TDEE following exercise, reflecting greater metabolic adaptation, will display distinct gut microbiome profiles characterized by lower abundance of SCFA-producing taxa and reduced microbial diversity.

## 2 Methods

### 2.1 Participants

Sixteen sedentary (<1-h regular exercise per week) men and women, aged 21–45 and overweight (BMI 25–30 kg/m^2^) were recruited to this single-arm clinical trial. Exclusion criteria included recent participation in other exercise or weight loss programs (<6 months), non-stable weight (>±3%), smoking, current or recent (<6 months) pregnancy, being postmenopausal, breastfeeding, having a history of weight loss surgery, or having cardiopulmonary conditions (e.g., recent myocardial infarction or unstable angina). Participants with musculoskeletal or neuromuscular impairments that would prevent exercise training, cognitive impairments, or those using chronic or metabolically active medications were also excluded.

### 2.2 Sample size

We estimated our sample size based on a previous intervention that examined the effect of aerobic exercise on gut microbiota composition over 6 weeks (31). That study reported a significant increase in the *Verrucomicrobia* phylum and a significant decrease in the Proteobacteria phylum. Using WinPepi software with *α* = 0.05 and *β* = 0.2 (i.e., 80% power), the minimum number of participants required to detect similar changes was calculated as 7 for *Verrucomicrobia* and 8 for Proteobacteria. To ensure sufficient statistical power while accounting for approximately 75% compliance with the intervention and 20% attrition, we included 16 participants in total.

### 2.3 Ethics

The study was approved by the Institutional Review Board of Sheba Medical Center (7214-20-SMC) and the ethics committee of Tel Aviv University (0001932-4). Informed consent was obtained from all participants prior to enrollment. The trial was registered at ClinicalTrials.gov (NCT04460040) and MyTrial.gov (7214-20SMC).

### 2.4 Physical activity intervention

All participants underwent moderate-intensity exercise (60–70% of their maximal rate of oxygen volume consumed during exercise, VO_2_ max) for 12 weeks. Exercise training was monitored using an optical heart rate sensor (Polar, OH1). The exercise intervention consisted of 3–5 weekly structured free-living walking sessions (group average: *250*–300 min/week) at moderate intensity with a weekly exercise energy expenditure target of 20 kcal/kg/week (1,500–2,000 kcal/week) for each participant. Participants completed one supervised treadmill exercise session per week in a WRIC within the laboratory, while the remaining sessions were conducted independently at home, in a gym, or outdoors. During the first 2 weeks, participants engaged in 150–200 min/week of moderate-intensity aerobic exercise to facilitate adaptation and reduce the risk of injury.

### 2.5 Dietary intake

Study participants were instructed to consume their habitual diet, and a 7-day dietary recall was used to monitor and confirm the stability of nutrient intake throughout the intervention. Dietary questionnaires were given for 7 days during the control phase (days −14 to −7), and during the last week of the intervention. Macro and micronutrient intake was calculated using “Nutratio”—an electronic food and nutrient database based on the Israeli Ministry of Health and USDA.

### 2.6 Anthropometry and body composition

Body weight was measured to the nearest 10 g with a digital scale (“mBCA” Seca) after a 12-h overnight fast on day −14, day 0, and once monthly throughout the intervention period. Height was measured at baseline with a wireless digital stadiometer to the nearest 0.1 cm. Waist and hip circumference were measured at day −14, day 0, and every month during the intervention period using a tape measure. Changes in body composition, including FM and FFM, were evaluated using the multi-frequency bioelectrical impedance analysis (BIA) technique and the “mBCA” body composition analyzer at day −14, day 0, and every month during the intervention. Recently, we have shown that body composition evaluation using this device is comparable to the reference method dual-energy X-ray absorptiometry (DXA) (32).

### 2.7 Total daily energy expenditure measurement

TDEE was measured over 10-day period at both baseline and at the end of the intervention using doubly labeled water (DLW), a gold-standard for assessing real-life energy expenditure. Before administrating the labeled water a baseline urine sample was collected to determine background levels of ^2^H and ^18^O. Participants then consumed an oral dose of water containing 1.8 g/kg total body water (TBW, estimated as 73% of FFM) of 10 atom percent excess ^18^O and 0.12 g/kg TBW of 99.9 APE ^2^H. Urine samples were subsequently collected 4 and 5 h after dosing. On day 10, participants were instructed to discard their first urine void of the day, then provided their second and third voids, which were collected in the lab. Sample aliquots (4 mL) were frozen at −80 °C pending analysis. Samples were later thawed, centrifugated and analyzed for ^18^O and ^2^H enrichment by off-axis integrated cavity output spectroscopy (OA-ICOS, Los Gatos Research Inc., Mountain View CA). Data was processed using commercially available Post Analysis Software (Los Gatos Research Inc., Version 2.2.0.12), which utilizes inter-run standard measurements to automatically calibrate isotope measurements. Samples were run in duplicate and repeated if the SD exceeded 2δ ^0^/00 for ^2^H and 0.5 ^0^/00 for ^18^O. Dilution spaces for ^2^H and ^18^O were calculated from the baseline samples following methods described by Prentice (33). Total body water was calculated as the average dilution spaces of ^2^H and ^18^O after correcting for isotopic exchange with other body pools. The CO_2_ production rate was determined using a modification of the original two-point equation (34). TDEE was calculated assuming a respiratory quotient of 0.86 and averaged over 10 days. All urine samples were collected in Dr. Gepner’s lab and were shipped for analysis to Dr. Melanson’s human metabolism lab at the University of Colorado. Adaptive TDEE is calculated by normalizing TDEE by the FFM, representing the daily energy expenditure per kilogram of lean mass (kcal/FFM/day) and accounting for individual differences in metabolically active tissue.

### 2.8 Resting metabolic rate measurement

RMR was measured using indirect calorimetry in an 11,500-liter whole-room indirect calorimeter (WRIC) at both baseline and the end of the intervention period. Each participant’s RMR was measured over 60 min after a 12-h overnight fast (7 p.m.–9 a.m.), ensuring that at least 24 h had passed from the last bout of any structured exercise and avoiding excessive exercise on the morning of testing. During the measurement, participants were instructed to remain motionless and awake in a supine position, lightly clothed and in a dimly lit, quiet and thermoneutral (22 °C–23 °C) room. Before each measurement, O_2_ and CO_2_ gas analyzers were calibrated using dry chemicals and, once per week, using standardized gas mixtures. The calorimeter’ air was released at 240 L/min. Oxygen uptake and carbon dioxide production were measured using gas analyzers (Promethion room calorimeter systems) and calculated using ExpeData software (Sable Systems, United States). The average of the final 20 min of the measurement was used to calculate RMR using Weir’s equation (EE = 3.9 × VO_2_ (L) + 1.1 × VCO_2_ (L)). Adaptive RMR is determined by normalizing RMR to FFM, reflecting the resting metabolic rate per kilogram of lean mass (kcal/kg FFM) and adjusting for individual variability in metabolically active tissue. Metabolic adaptation defined as the differences between measured and predicted changes in energy expenditure (typically RMR or TDEE) relative to changes in fat-free mass and fat mass, reflects the compensatory response to the exercise intervention.

### 2.9 Stool sample collection, processing, and sequencing

Samples were collected pre- (a week before) and post- (during last week) intervention, alongside bowel activity status and stool quality questionnaires. Each subject received two stool test tubes at each time point along with specific instructions for collecting the stool and storing it immediately in their home freezers, pending transfer on dry ice to the laboratory freezer (−80 °C). During analysis, the stool samples were thawed and subjected to DNA extraction. Extracted DNA samples were quantified using the GloMax Plate Reader System (Promega) and QuantiFluor^®^ dsDNA System (Promega) chemistry. DNA libraries were prepared using the Nextera XT DNA Library Preparation Kit (Illumina) and were quantified using Qubit 4 fluorometer and Qubit^™^ dsDNA HS Assay kit. The libraries were then sequenced on an Illumina NovaSeq 6000 platform at 2× 150 bp.

### 2.10 Metagenomic quality control and annotation

Shotgun metagenomics reads went through quality trimming and adapter removal using fastp, and host DNA reads were filtered by aligning reads to the human reference genome GRCh38 (35) using Bowtie 2 (36). Reads that passed these QC steps were classified taxonomically by k-mer classification using Kraken2 (37) against a GTDB (38) representative species database. Finally, the Bracken (39) pipeline was run on the Kraken taxonomic classification output to estimate species-level relative abundances within each sample.

### 2.11 Alpha and beta-diversity measures calculation

Taxonomic variation within and between samples was calculated based on the Bracken species-level relative abundance tables, in comparison to the reference GTDB v207 phylogenetic tree. Bray–Curtis dissimilarity, weighted unique fraction (UniFrac), and Shannon diversity indices were calculated using the Phyloseq (40) package, and Faith’s phylogenetic diversity (PD) index was calculated using the Picante (41) package in R 4.1.2. Beta-diversity distances were computed between each pair of samples, and principal coordinates analysis (PCoA) was used to examine these distances across response groups.

### 2.12 Definition of exercise training response groups

Subjects were categorized as exercise training responders vs. non-responders according to the relative change in FM and FFM during the training period. Specifically, we considered response as reflecting a general improvement in the body composition, based on the notion that FFM increase, and FM decrease are both positive outcomes (42). Accordingly, responders were defined as participants for whom the change in FFM was greater than the change in FM: (FFMPost − FFMPre) − (FMPost − FMPre) >0.

### 2.13 Statistical analysis

Response groups were tested for possible confounding demographic and pre-treatment anthropometric properties. Fisher’s exact test was employed to examine gender distribution between the response groups, and Wilcoxon rank-sum test was used to evaluate differences in age and pre-training BMI, FM, and FFM. Post multiple-testing correction (FDR) results indicated there were no significant differences in these properties between the response groups, confirming that the groups were well-balanced at baseline regarding potential confounders. When analyzing microbiome-associated features such as differential abundance, diversity metrics and functional pathway analysis we used the Wilcoxon rank-sum test when comparing response groups, and the paired Wilcoxon signed-rank test when comparing longitudinal changes within individuals within each group. Functional pathway abundances were profiled using HUMAnN2 (43), filtered for prevalence (>10% of samples), and normalized to relative abundance.

### 2.14 Response prediction by pre-training GM taxonomic profiles

A subset of 245 species were selected for relative abundance (RA) analysis based on minimal abundance (mean RA) >0.05% and prevalence (detection in at least 80% of samples) in the pre-training samples. These species’ RA values were utilized to construct a series of univariate logistic regression models predicting the post-training response label. Reported receiver operating characteristic (ROC) curve AUC (AUROC) values are the result of k-fold cross validation, generated with the rsample, glm, and pROC packages, and were used to determine highly informative species (AUROC >0.8)^.^ To ensure the reliability of these predictors, we also employed multivariate regularized regression and calculated *p*-values for each ROC curve using a Mann–Whitney approach (44), adjusting for multiple testing (FDR <0.1, 95% CI >0.5).

### 2.15 Code and data availability

Code for data preparation and analysis is available at https://github.com/borenstein-lab/microbiome_metabolic_adaptation. Data will be made available on request of the corresponding author.

## 3 Results

### 3.1 Metabolic changes following moderate intensity exercise training

**Figure 1:**
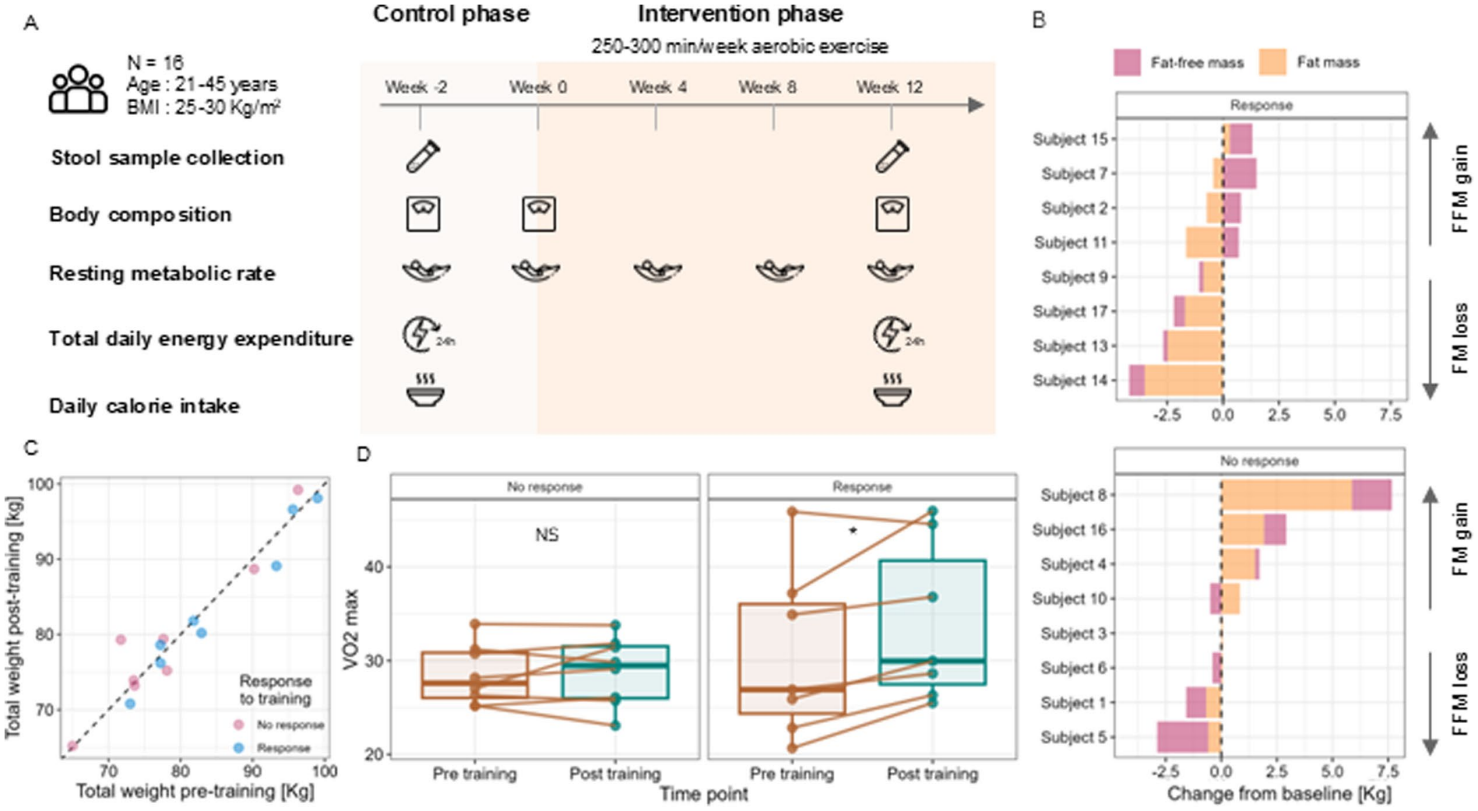
Study design for linking metabolism, body weight measures, and the gut microbiome. **(A)** An overview of the study design. A cohort of 16 sedentary overweight adults underwent 12 weeks of moderate-intensity physical activity (PA), with stool sample collection, body composition, RMR and TDEE measurement, and food questionnaires filled pre-training (week −2) and post-training (week 12) time points. **(B)** Response to exercise training is defined by the relative change in fat mass (FM, pink) and fat-free mass (FFM, yellow) during the training period, with responders having the change in FFM being greater than the change in FM, and vice versa for non-responders. **(C)** Subject-level total weight measurements pre- and post-training are not significantly different in either response (blue) or non-response (pink) groups (paired Wilcoxon signed-rank test, *p* = 0.236 and *p* = 0.46, respectively). **(D)** Maximal oxygen consumption (VO_2_ max) of subjects post-training is increased only in responders to exercise training (paired Wilcoxon signed-rank, *p* < 0.05). Icon made by Freepik from www.flaticon.com.

Sixteen healthy individuals, with a mean age of 38.9 ± 3.7 years and mean weight of 81.7 ± 10.2 kg, completed this study (Figure 1A). Despite high adherence (95%), the participants’ body weight remained unchanged (0.1 ± 2.1 kg, NS, Wilcoxon test) with no significant changes in anthropometric measures or body composition.

Absolute TDEE increased post-intervention (194 ± 304 kcal/day, *p*-value = 0.034, Supplementary Table S1), but TDEE per FFM (adaptive TDEE) showed no significant change (2.9 ± 5.9 kcal/kg/day, NS). Moreover, RMR per FFM (adaptive RMR) significantly decreased, implying improved energy efficiency. Furthermore, reported energy intake remained stable during the intervention (as confirmed by the lack of significant change in caloric intake in either response group, Supplementary Figure S1), suggesting that exercise alone may not be sufficient for net reduction in body weight.

### 3.2 Anthropometric and aerobic capacity improvements in responders to exercise

Although group-level body composition remained unchanged, high intra-individual variability was observed. Applying response groups classification showed that half of the participants (*n* = 8) improved their body composition, while the others showed no changes or worsening (Figure 1B). Despite no differences in total body weight between the groups post-training (Figure 1C), only responders exhibited a significant increase in VO₂max (Figure 1D). This improvement was observed despite there being no baseline differences between the groups in key potential confounders including gender, age, mean exercise time, caloric intake, and pre-training FM, FFM, and BMI (Supplementary Figure S1).

### 3.3 Response prediction by GM species relative abundance

**Figure 2:**
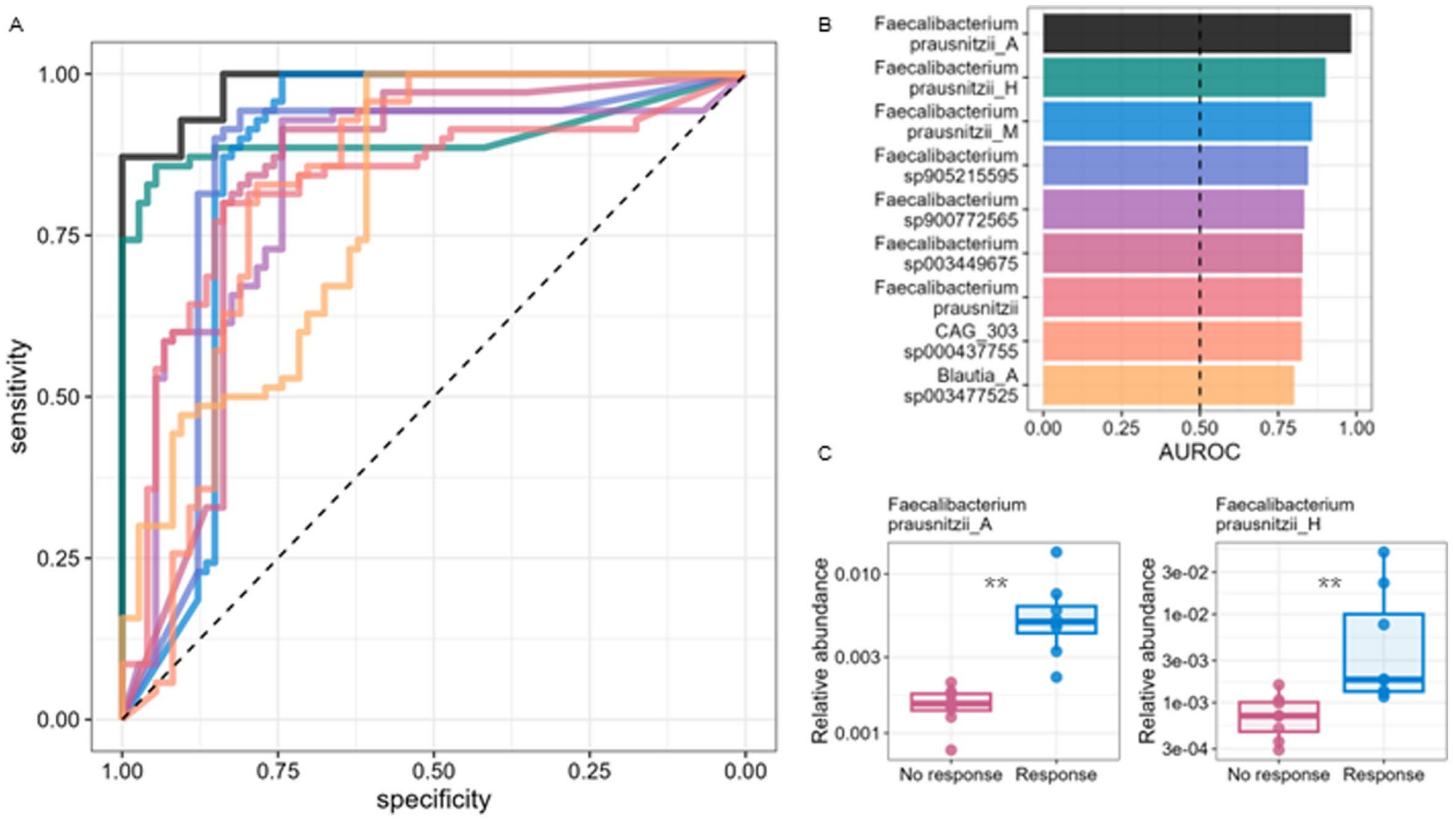
Response prediction based on microbiome species abundance pre-training. **(A)** Receiver operating characteristic (ROC) curve for response prediction by univariate logistic regression models and species pre-training relative abundance values. Each colored line represents a “highly predictive” species, defined as having ROCAUC >0.8 in 10-repeat *k*-fold cross-validation (*k* = 3). These species were selected out of a group of 245 species found to have sufficient prevalence pre-training (identified in at least 80% of samples, with minimal mean relative abundance of 0.05%). **(B)** Taxonomic names and ROCAUC values of highly predictive species, with the dashed line marking ROCAUC values expected by a random predictor. Six of these taxa are labeled as *Faecalibacterium prausnitzii* species, while the remaining three taxa belong to the butyrate-producing genera of the *Faecalibacterium*, *Roseburia* and *Blautia*. **(C)** Pre-training relative abundance values of the top two highly predictive species, split by response group. The abundance of all highly predictive species is significantly higher in responders (Wilcoxon rank-sum test, FDR adjusted *p* < 0.01).

To evaluate the capacity of taxa abundance to predict response, we constructed univariate logistic regression models using the baseline abundance of 245 prevalent species (Methods). Predictive power was quantified by AUROC, with 10-repeat 3-fold cross-validation to maintain class balance. We defined “highly-predictive” species as having AUROC >0.8 (Figure 2A), with *F. prausnitzii* species making up six of the nine highly-predictive species (Figure 2B, Methods). Overall, *F. prausnitzii* species AUROC values were significantly higher than those obtained for other species (0.76 ± 0.13 vs. 0.65 ± 0.08, Wilcoxon rank-sum test, *p*-value = 0.0008). The remaining highly-predictive taxa include unassigned species of the *Faecalibacterium*, *Roseburia* and *Blautia* genera, known butyrate producing genera associated with gut health (Figure 2C; Supplementary Figure S2). The robustness of these species was further confirmed through multivariate regularized regression and significance testing with multiple-testing correction (see Methods, Supplementary Figure S3).

Overall, a significant association between increased abundances of specific GM species and body composition improvement following the exercise intervention. Interestingly, several predictive species—*Faecalibacterium_900772565*, *Roseburia_CAG-303_sp000437755*, and *Blautia_A_sp003477525*, were not previously discussed in this context, suggesting novel microbial contributors to training effectiveness. We also examined microbial functional potential via metabolic pathway analysis, but found no statistically significant differential abundance between groups or over time after correcting for multiple testing (FDR >0.1; Supplementary Table S2).

### 3.4 Intra-sample diversities separate response groups pre- and post-training

We next investigated the relationship between exercise response and community-wide GM properties- alpha and beta-diversity.

**Figure 3:**
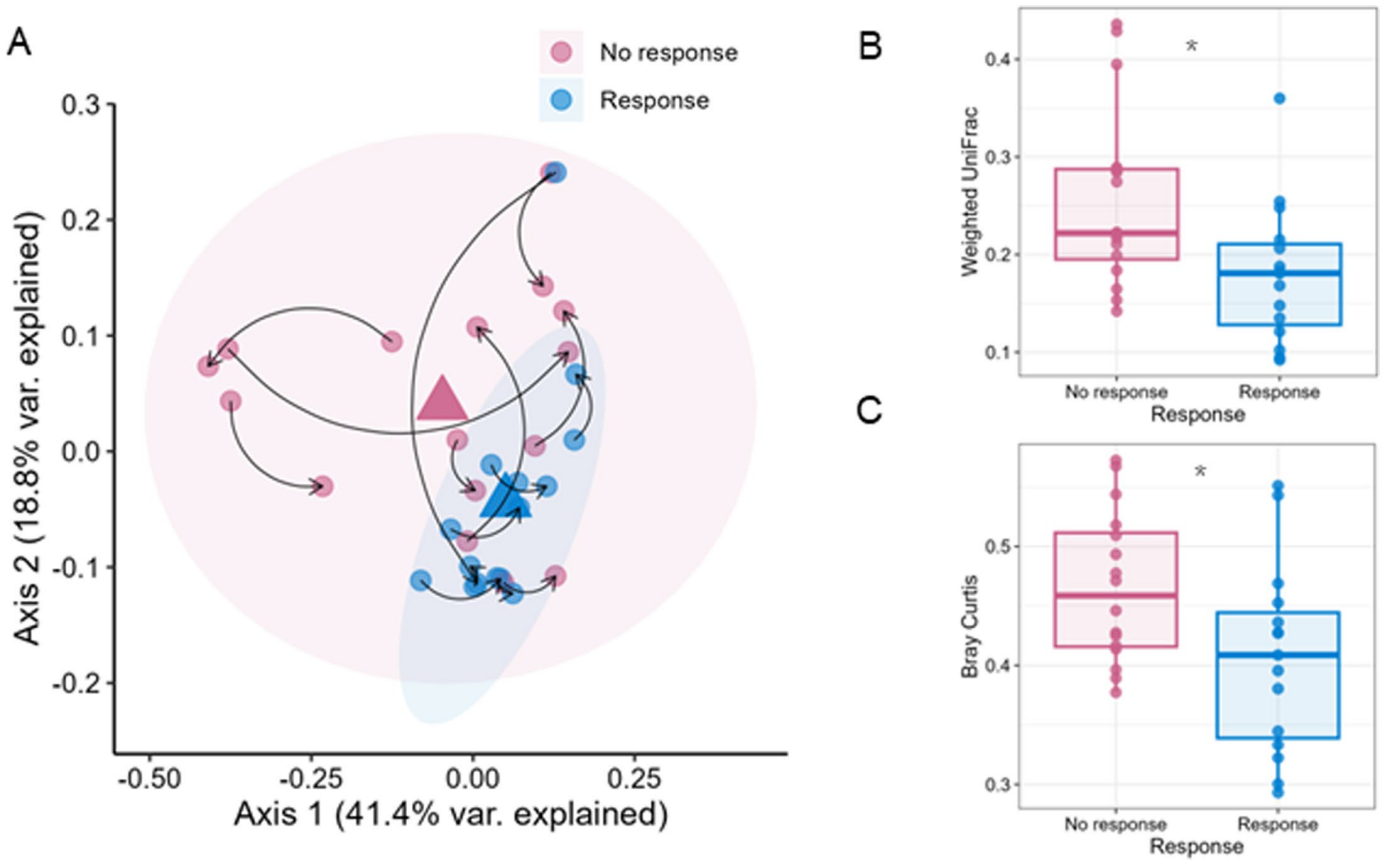
Intra-sample distances (beta diversity) between response groups. **(A)** Principal coordinates analysis (PCoA) of the first two principal coordinates based on weighted UniFrac distances of all the cohort samples, colored by response (blue) and non-response (pink) groups, with arrows linking pre-to-post training samples of the same subject, with group outlines (ellipses) and centroids (triangles) marked with corresponding colors. Samples of response and non-response groups have significantly different dispersion (dispersion test, *p* = 0.017) and centroid location (PERMANOVA test, *p* = 0.024, 9,999 permutations). Comparison of Euclidean distances in principal coordinate space between the samples and their respective group centroid for **(B)** weighted UniFrac and **(C)** Bray–Curtis beta diversity measures (Wilcoxon rank-sum test, *p* < 0.05).

We found that responder samples across time points formed a cohesive group, whereas non-responder samples were more dispersed (Figure 3A; Supplementary Figure S4A). Differences in centroid location (mean) and dispersion (variance) were quantified using a permutational multivariate analysis of variance (PERMANOVA) test, revealing significant differences between response groups (weighted UniFrac *p*-value = 0.024, Bray–Curtis *p*-value = 0.009). Notably, within-group variances significantly differ across response groups in both beta-diversity measures, as determined by the multivariate homogeneity of group dispersions test (weighted UniFrac *p*-value = 0.017, Bray–Curtis *p*-value = 0.029). Finally, responder samples are significantly closer to their group centroid in the multi-dimensional PCoA space (Figures 3B,C, Wilcoxon rank-sum test, *p*-values are 0.01 and 0.04, respectively). Interestingly, distances from pre- to post-training samples were smaller in responders, although this did not reach statistical significance.

### 3.5 RMR and TDEE adaptation association with species richness dynamics

**Figure 4:**
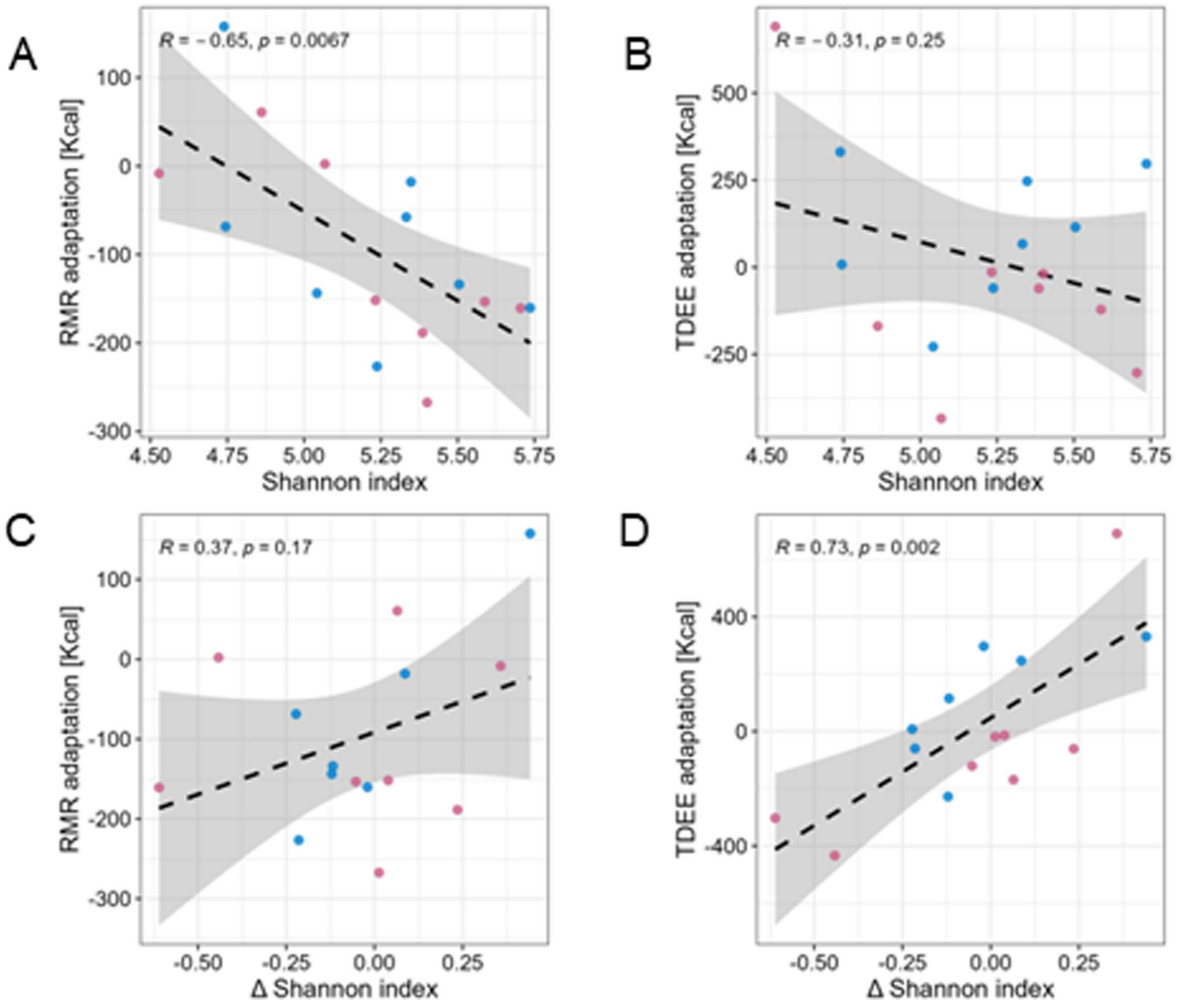
Correlation between metabolic adaptation and inter-sample diversity (alpha diversity). Pearson correlations between Shannon index values of pre-training samples and RMR **(A)** and TDEE **(B)** adaptation following exercise training, showing a negative trend. In contrast, a positive correlation is found between the change in Shannon index and these metabolic adaptations (**C,D**, respectively), indicating that an increase in alpha diversity is associated with increased energy expenditure post-training.

To further investigate the relationship between GM community structure and metabolic adaptation, we tested the correlation between pre-training alpha-diversity (Shannon index) and changes in RMR and TDEE post-intervention. Pre-training alpha-diversity was negatively correlated with RMR adaptation (Pearson’s *R* = −0.65, *p* = 0.0067), meaning that subjects with highly diverse GM population pre-training tended to reduce their resting energy expenditure (Figure 4A), regardless of response classification. A similar, though non-significant trend exists with TDEE adaptation (Figure 4B). We then examined the association between *changes* in alpha-diversity and metabolic adaptation, revealing a strong positive correlation between an increase in alpha-diversity and TDEE adaptation (Pearson’s *R* = 0.73, *p* = 0.002, Figure 4D)—i.e., subjects whose GM became more diverse also increased their energy expenditure (and to a lesser degree their RMR, Figure 4C). In contrast to the metabolic metrics, we observed no significant correlation between alpha-diversity and VO_2_ max at baseline or in response to the intervention (Supplementary Figures S4B,C).

## 4 Discussion

This study examined the relationship between GM composition, moderate aerobic exercise, and metabolic adaptation in sedentary overweight adults. Given the inherent variability in individual responses to exercise, participants were classified into response groups, based on individual changes in body composition post-intervention. Interestingly, beyond enhanced body composition, responders demonstrated an improved aerobic capacity compared to non-responders. We followed up with a comprehensive analysis of GM pre- and post-training metagenomic samples, examining both species-level association and broader community structure. By integrating these microbial profiles with metabolic data, we aimed to identify gut microbiome characteristics that may explain individual variability in metabolic adaptation—defined here as the discrepancy between measured and predicted changes in energy expenditure relative to body composition changes.

Analysis of GM composition pre-training revealed that certain species were highly predictive of response, including several *F. prausnitzii* species and other species unreported previously in this context, which could potentially be beneficial to effective response to exercise training. Responders further displayed a more cohesive GM structure (in terms of their beta-diversity), whereas non-responders exhibited greater variability across individuals. While the cohort overall exhibited metabolic adaptation (improved energy efficiency via decreased adaptive RMR), this response was not uniform. Interestingly, metabolic adaptation was negatively correlation with pre-training alpha-diversity and positively correlated with the *change* in alpha-diversity post-training (Figure 4). This suggests that although the general physiological tendency was toward energy conservation, participants with lower diversity pre-training or greater gains in diversity during the raining period were more likely to increase their energy expenditure. This study highlights the strong role of the GM on exercise-induced changes in human body composition and energy metabolism.

We defined response as a relative changes in body composition rather than absolute reduction in BMI, as this measure does not necessarily imply improvements in physical health. Evidence suggests that elevated FM is associated with increased cardiovascular risk and mortality, independent of body weight (45). For example, BMI reduction driven by FFM loss worsens the body composition by effectively increasing %FM. Conversely, a weight from increased FFM can represent a positive physiological change.

The strong predictive power of *F. prausnitzii* underscores its potential as a biomarker for exercise-related body composition changes, consistent with its known anti-inflammatory effects in gut health (46, 47). Additionally, we identified three highly predictive species (Figure 2B) from the *Faecalibacterium*, *Roseburia*, and *Blautia* genera. Given their predictive power, these taxa warrant further investigation to clarify their role in exercise-related body composition changes and overall health.

Metabolic compensation is a behavioral and physiological adjustments reducing the effects of changes in activity or diet. Common in exercise interventions, though their degree varies among individuals and studies, making exercise alone a poor predictor of TDEE. However, many weight-loss studies do not measure TDEE directly, limiting insight into the interplay between exercise, energy intake, and TDEE changes.

In our study, we observed a significant negative correlation between pre-training alpha-diversity and RMR adaptation. Furthermore, a significant decrease in adaptive RMR [kcal/FFM(kg)] was observed. As discussed above, higher alpha-diversity is associated with lower levels of pro-inflammatory markers (48, 49), thus reducing the metabolic cost associated with inflammation, potentially lowering RMR, and affecting the multifaceted mechanism of metabolic compensation.

This study has several limitations that should be acknowledged, notably the modest sample size and the single-arm design which limit the causal conclusions of this study. Given the high dimensionality of microbiome data relative to this cohort size, the predictive models identified here should be interpreted with caution as hypothesis-generating results. However, the primary aim was to investigate the inter-individual variability in metabolic and microbial adaptation following a standardized exercise dose. Findings should be validated in a larger multi-arm cohort with additional temporal sampling to establish response trajectories and ensure robustness before any clinical or translational implications are drawn. Moreover, species were linked to response based on their baseline abundance, and while these associations are robust, determining causality requires further validation. Future mechanistic studies, employing metabolomics or *in vivo* models, are required to confirm these links and explain the microbiome’s specific role in metabolic adaptation.

Despite these limitations, this study’s strengths include rigorous measurement of physiological and behavioral aspects of participants using gold-standard techniques. Indeed, our analysis has identified three novel candidate species suggested to be beneficial for an effective response to exercise, supported by the identification of known *F. prausnitzii* species.

Additionally, we identified microbiome community states more consistently associated with response, while non-responders exhibited a wider range of states. We also established a relationship between taxonomic diversity and energy expenditure adaptation (RMR and TDEE), demonstrating that both metrics shift in a positively correlated manner following the intervention.

In conclusion, our findings provide important insights into the intricate relationship between exercise, metabolism, and the GM. Understanding effective responses to exercise and the mechanisms of metabolic compensations is crucial for addressing the healthcare challenges posed by metabolic diseases.

## Data Availability

The original contributions presented in the study are included in the article/Supplementary material, further inquiries can be directed to the corresponding authors.

## Supplementary material

SUPPLEMENTARY FIGURE S1 Pre-training potential metadata confounders between response groups. Inspection of potential confounders revealed no significant differences between response groups (FDR adjusted *p*-values of Fisher’s exact test for gender and Wilcoxon rank-sum test for age, BMI, FFM, FM, exercise time, and caloric intake).

SUPPLEMENTARY FIGURE S2 Pre-training relative abundance differences of highly predictive species between response groups. Pre-training relative abundance values of the highly predictive species, split by response group. The abundance of all highly predictive species is significantly higher in responders (Wilcoxon rank-sum test, FDR adjusted *p*-values; ^*^*p* < 0.05 and ^**^*p* < 0.01).

SUPPLEMENTARY FIGURE S3 Robustness analysis of highly-predictive species. **(A)** Feature coefficients from a multivariate regularized logistic regression model (Elastic Net) trained on the full dataset to predict response. Species with positive coefficients (indicating higher abundance in responders) include eight of the nine species originally identified as “highly predictive” (colored bars), alongside additional taxa (grey bars). **(B)** Receiver operating characteristic (ROC) AUC values for species demonstrating significant predictive performance (Mann–Whitney test, FDR <0.1). Species are ranked by AUC with 95% confidence intervals (error bars), where point size scales with significance [−log10(FDR)]. Colored points indicate the original “highly predictive” species, while grey points mark other significant species.

SUPPLEMENTARY FIGURE S4 Beta diversity between response groups and alpha diversity-VO_2_ max correlation**. (A)** Principal coordinates analysis (PCoA) of the first two principal coordinates, as in Figure 3A, but based on Bray–Curtis distances (instead of weighted UniFrac) of all the cohort samples, colored by response (blue) and non-response (pink) groups, with arrows linking pre-to-post training samples of the same subject, with group outlines (ellipses) and centroids (triangles) marked with corresponding colors. **(B,C)** Scatter plots showing the correlation between VO_2_ max and alpha diversity (Shannon index) at baseline **(B)** and between the changes (delta) in these metrics **(C)** (Pearson correlation).

SUPPLEMENTARY TABLE S1 Energy expenditure components and weight loss change. Comparison of energy expenditure components change between baseline and post intervention periods. Adaptive change refers to alterations based on FFM. Total daily energy expenditure (TDEE), resting metabolic rate (RMR), sleeping metabolic rate (SMR), diet induced thermogenesis (DIT), kilograms (kg), FFM (fat free mass), kilo calories (kcal), weight change. All data are reported as mean ± SD. Group comparison was performed using the Mann–Whitney test.
