# Supplementary figures and images for: The associations between physical activity, microbiome and metabolic adaptation in sedentary overweight adults
Source: Front Nutr. 2026 Feb 2;12:1722274. doi: 10.3389/fnut.2025.1722274 (PMC12908587; doi:10.3389/fnut.2025.1722274)

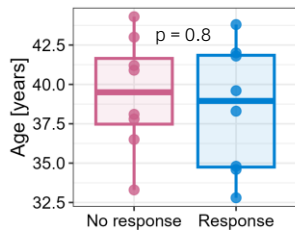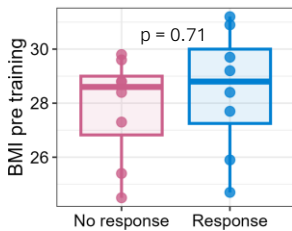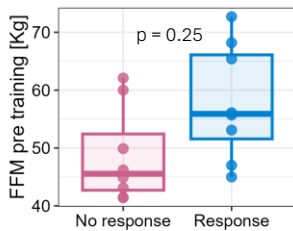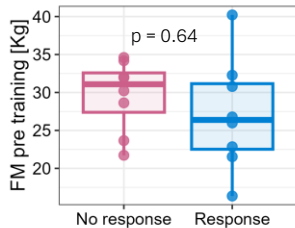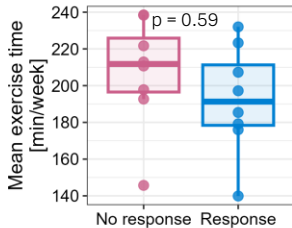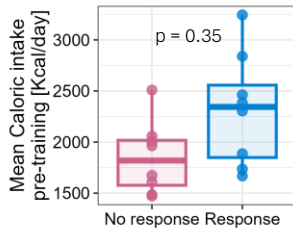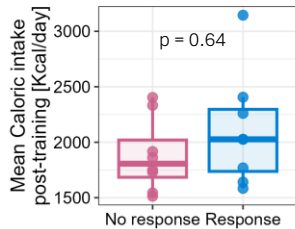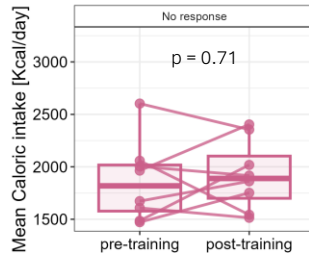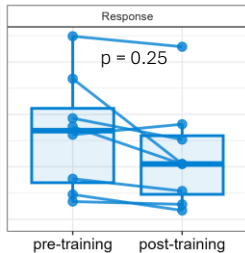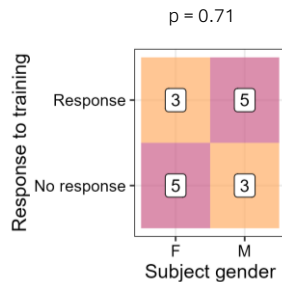

Supplement: SUPPLEMENTARY FIGURE S1 — Pre-training potential metadata confounders between response groups. Inspection of potential confounders revealed no significant differences between response groups (FDR adjusted p-values of Fisher’s exact test for gender and Wilcoxon rank-sum test for age, BMI, FFM, FM, exercise time, and caloric intake). [file Image_1.pdf]

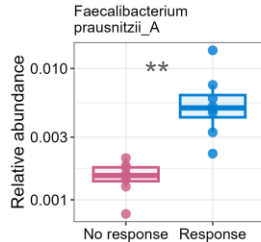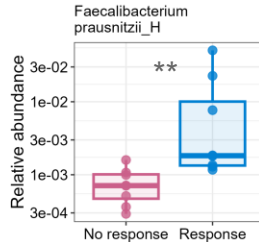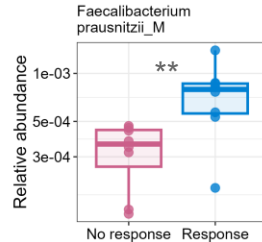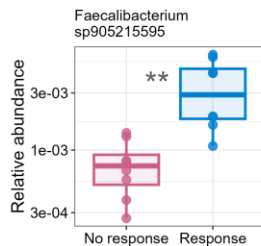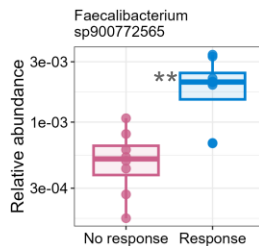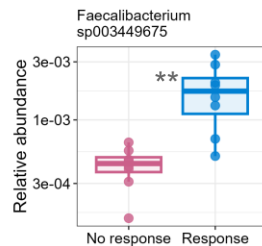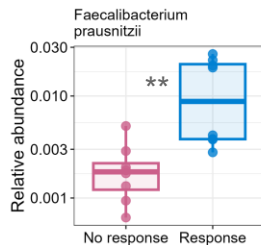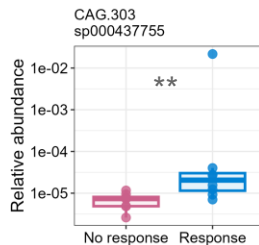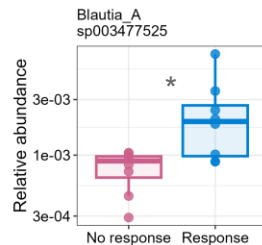

Supplement: SUPPLEMENTARY FIGURE S2 — Pre-training relative abundance differences of highly predictive species between response groups. Pre-training relative abundance values of the highly predictive species, split by response group. The abundance of all highly predictive species is significantly higher in responders (Wilcoxon rank-sum test, FDR adjusted p-values; *p < 0.05 and **p < 0.01). [file Image_2.pdf]

A

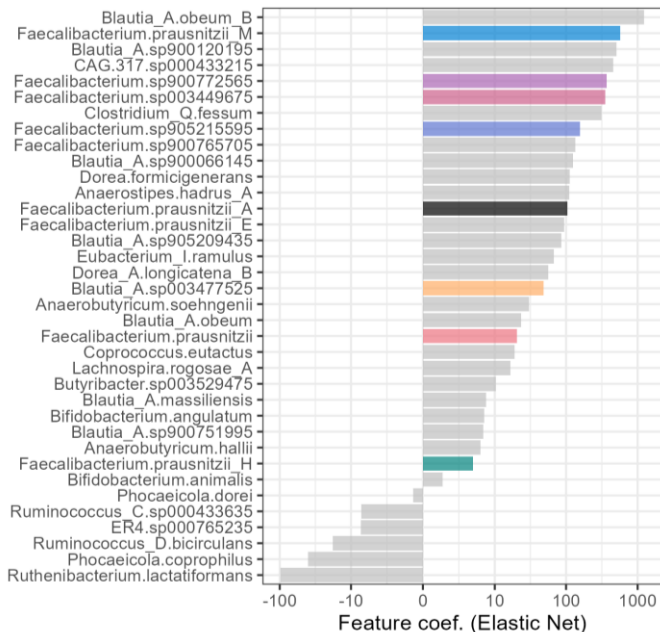

B

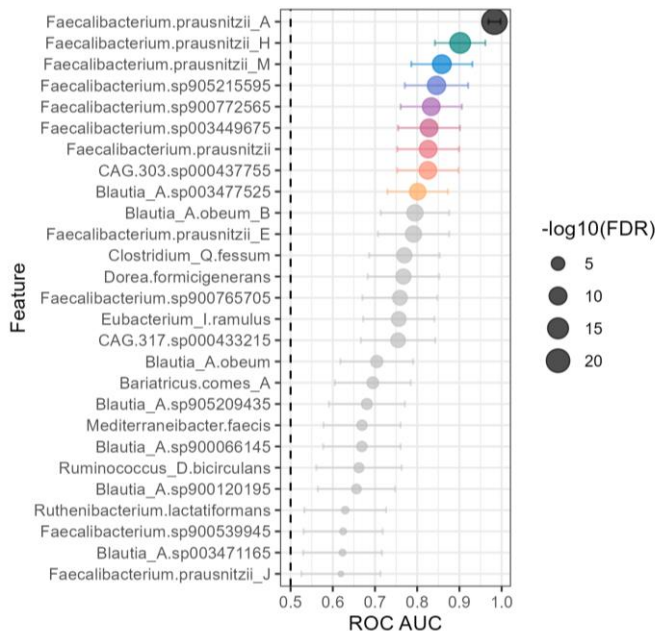

Supplement: SUPPLEMENTARY FIGURE S3 — Robustness analysis of highly-predictive species. (A) Feature coefficients from a multivariate regularized logistic regression model (Elastic Net) trained on the full dataset to predict response. Species with positive coefficients (indicating higher abundance in responders) include eight of the nine species originally identified as “highly predictive” (colored bars), alongside additional taxa (grey bars). (B) Receiver operating characteristic (ROC) AUC values for species demonstrating significant predictive performance (Mann–Whitney test, FDR <0.1). Species are ranked by AUC with 95% confidence intervals (error bars), where point size scales with significance [−log10(FDR)]. Colored points indicate the original “highly predictive” species, while grey points mark other significant species. [file Image_3.pdf]

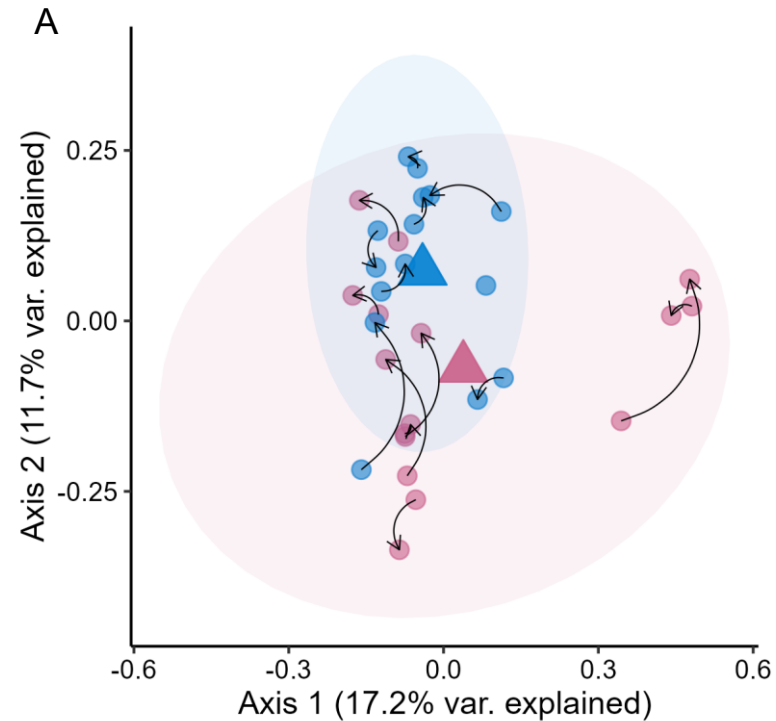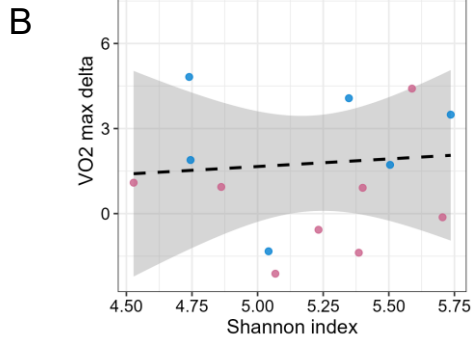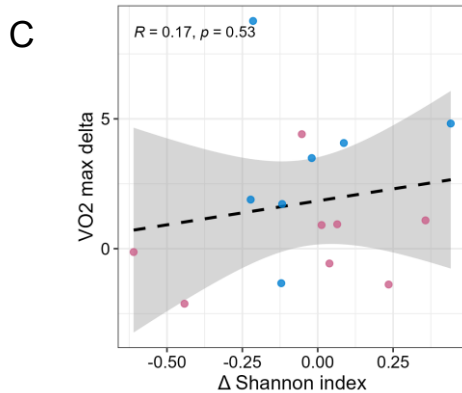

Supplement: SUPPLEMENTARY FIGURE S4 — Beta diversity between response groups and alpha diversity-VO2 max correlation. (A) Principal coordinates analysis (PCoA) of the first two principal coordinates, as in Figure 3A, but based on Bray–Curtis distances (instead of weighted UniFrac) of all the cohort samples, colored by response (blue) and non-response (pink) groups, with arrows linking pre-to-post training samples of the same subject, with group outlines (ellipses) and centroids (triangles) marked with corresponding colors. (B,C) Scatter plots showing the correlation between VO2 max and alpha diversity (Shannon index) at baseline (B) and between the changes (delta) in these metrics (C) (Pearson correlation). [file Image_4.pdf]
